# Trees outside forests are an underestimated resource in a country with low forest cover

**DOI:** 10.1038/s41598-021-86944-2

**Published:** 2021-04-12

**Authors:** Nathan Thomas, Priscilla Baltezar, David Lagomasino, Atticus Stovall, Zaheer Iqbal, Lola Fatoyinbo

**Affiliations:** 1 Earth System Science Interdisciplinary Center, University of Maryland, College Park, MD 20740 USA; 2 Biospheric Sciences Laboratory, NASA Goddard Space Flight Center, Greenbelt, MD 20771 USA; 3 Department of Coastal Studies, East Carolina University, Wanchese, NC 27981 USA; 4 Bangladesh Forest Department, Resource Information Management System (RIMS), Dhaka, 1207 Bangladesh

**Keywords:** Forestry, Environmental sciences

## Abstract

Trees outside forests (TOF) are an underrepresented resource in forest poor nations. As a result of their frequent omission from national forest resource assessments and a lack of readily available very-high-resolution remotely sensed imagery, TOF status and characterization has until now, been unknown. Here, we assess the capacity of openly available 10 m ESA Sentinel constellation satellite imagery for mapping TOF extent at the national level in Bangladesh. In addition, we estimate canopy height for TOF using a TanDEM-X DEM. We map 2,233,578 ha of TOF in Bangladesh with a mean canopy height of 7.3 m. We map 31 and 53% more TOF than existing estimates of TOF and forest, respectively. We find TOF in Bangladesh is nationally fragmented as a consequence of agricultural activity, yet is capable of maintaining connectedness between remaining stands. Now, TOF accounting is feasible at the national scale using readily available datasets, enabling the mainstream inclusion of TOF in national forest resource assessments for other countries.

## Introduction

Trees outside forests (TOF) are an overlooked and undervalued resource that are not regularly included in national inventories. Subsequently, data on TOF extent and structure is often scarce, despite their increasing importance in a continually urbanizing world. The continued rate of urbanization worldwide is driving an increase in concern for the separation of people from the natural environment^1^ and TOF will be heavily relied upon as surrogates for natural forests and the services that they provide, albeit to a more limited capacity than large intact forests^2^. This is pertinent where TOF may provide the only wood resources to local populations^3,4^, as in developing nations where two-thirds of fuel wood is derived from TOF^5^. Furthermore, at a national level TOF are capable of contributing significantly to biomass stocks^6^ but are overlooked in rural or agricultural areas despite 40% of all global agricultural land containing a tree cover of more than 10%^7^. This has been observed in South Asia whereby production from TOF has not been fully realized, despite the potential of TOF at the national level to decrease the gap between timber demand and supply^8^. Including TOF in resource accounting is therefore paramount to achieving accurate data on wood volume stocks, and ensuring the provision of ecosystem services to rural populations.

The lack of inclusion of TOF in forest assessments has led to little knowledge of TOF across large areas, especially at the national reporting level. The United Nations Food and Agricultural Organization (UN FAO) introduced TOF into their Global Forest Resource Assessment (FRA) of 2000^9^. The FAO definition of TOF are trees that occur outside of the FAO definitions of forest and other wooded land (OWL)^10^, yet TOF do not definitively adhere to limitations on extent nor crown cover^11^. Subsequently in 2005 the FAO added a category of Other Land with Tree Cover (OLwTC) to the RFA to account for forests that meet the FAO forest definitions but are situated within a predominantly agricultural or urban setting^12^. Here we adopt this definition of TOF. This category was easier to implement in practice and enabled all tree resources to be included in national forest inventories, thus providing a pathway for the inclusion of TOF. This was bolstered by a range of UN initiatives that required the inclusion of TOF to meet adequate levels of reporting (e.g., UNFCCC)^13^. In addition, inventories conducted under the National Forest Monitoring and Assessment (NFMA) program of the UNFAO include all trees irrespective of land use^14^ and this approach has also been adopted by a limited number of national forest inventories that include TOF in their reporting^15^. However, the monitoring of TOF is highly variable, both in terms of the level of inclusion of TOF and the quality of reporting. Bias exists in the inclusion of trees in one domain and not in others, such as in agricultural land but not in urban environments, further exacerbated by additional barriers such as insufficient land access. Moreover, even when included in exhaustive inventories, the data is not guaranteed to be publicly available^2^.

The adoption of a participatory approach to afforestry in Bangladesh, has seen the movement of people from shifting cultivators, with vulnerable livelihoods to securing financial income, resources and employment from homestead and community forestry^16^. This has increased the importance and reliance upon TOF, particularly in rural areas where TOF have become a vital source of timber, fuel, food and income^17,18^ whilst at the national level accounts for in excess of 70% of total aboveground tree biomass^2^. In response to the UN FAO Forest resources Assessment^19^ that approximated a total forest cover of 1.5 million ha, equitable to 0.009 ha of forest per person, the quantity of planted trees from household to industrial scales, has increased. The Bangladesh forest policy of 1994, for the first time, institutionalized participatory social forestry and promoted both the cultivation and worth of TOF. This was driven by government policy that promoted participatory social forestry and it has been successful primarily due to committed parties at the local level. At the time, the Bangladesh government was not suitably placed to implement international forest policies and most of the beneficial progress since has been observed at the grassroots level^20^. More recently the Bangladesh Forest Department have conducted National Forest Inventories (NFI’s) and were set to finalize their most recent NFI in 2018, with support from the FAO. Until this, the structure of TOF (e.g., height) was unknown, leaving a knowledge gap in the vertical dimension. In an open agricultural landscape such as in Bangladesh, TOF provide an additional third dimension which has important ecological implications, such as maintaining connectivity between species which is not possible in agricultural areas alone. The ecological importance of TOF is therefore disproportionately important given the land area that it occupies^21^. Given the spatially continuous agricultural land use in Bangladesh, we adopt the FAO definition of Other Land with Tree Cover (OLwTC)^12^ as our definition of TOF.

Remote sensing has been an underused tool in the monitoring of TOF, yet is able to complement field based forest assessments and inventories by mapping the spatially continuous distribution and variability in stand size and structure, at the national level. Remote sensing has traditionally been used in tandem with field data to attain spatially explicit information on stand characteristics and reduce the uncertainty of population parameter estimates as a result of sampling, but this is less feasible with the cost of sufficiently high-resolution data needed to map TOF. This is driven by the mismatch in scales between large area coverage and the resolution required for mapping TOF, further constrained by the accuracy needed by Payment for Ecosystem Services (PES) schemes (e.g., REDD+). A limited number of studies have utilized remote sensing, with the most recent and thorough study estimating that tree canopy cover area in Bangladesh accounts for approximately 21.5% (3,165,550 ha) of the total area of Bangladesh, increasing by 135,700 ha between 2000 and 2014^22^. This work provided wall-to-wall estimates of TOF extent and cover, but at a resolution which is often at or below the patch size of TOF. The use of very-low-resolution imagery (e.g., MODIS, 500 m) for monitoring TOF are scarce, with the number of studies increasing with increasing resolution, particularly with active sensors such as LiDAR^23–25^. Such data, however, is time-consuming to collect and process, limiting repeated studies and on-going monitoring efforts. If resolution is therefore a constraining factor of remote sensing for TOF, recently available higher-resolution satellite imagery from the ESA Copernicus constellation, offer the potential to bridge the gap between satellite observations and field data for national scale resource inventories. TOF has yet to be mapped using radar imagery, despite its use in mapping and quantifying other forest attributes^26–28^, thus providing a potentially resourceful yet unexplored dataset for mapping TOF. With a suite of higher-resolution high-cadence publicly available datasets through ESA, it is worthwhile assessing the potential increased benefits that these sensors can provide.

Despite the routine estimation of forest height metrics from both airborne and terrestrial LiDAR systems^29–31^, this data is rarely available at the national scale. Globally available elevation data is in excess of 30 m resolution (e.g., SRTM) and estimates surface height above a reference geoid, which is unable to estimate tree height directly where the ground surface cannot be determined. Acquiring maps of canopy height would be unparalleled for informing national resource assessments, particularly for TOF which are composed of small stands covering large geographical areas, leading to time intensive field surveys. The ability to gain an accurate estimation of extent and height of trees at a national level would allow further integration with national resource accounting and increase a nation’s capacity for monitoring its tree resource.

We used globally available 10 m Sentinel-1 radar and Sentinel-2 optical satellite imagery to map TOF extent for Bangladesh using the Google Earth Engine. National scale mosaics were generated and thresholds were used on image values and indices to detect photosynthetic active land cover with aboveground structure. Canopy height was derived from a 30 m TanDEM-X Digital Surface Model (DSM), by combining the difference between the DSM and a derived interpolated Digital Terrain Model (DTM) with the TOF extent.

## Results

### Tree extent

**Figure 1:**
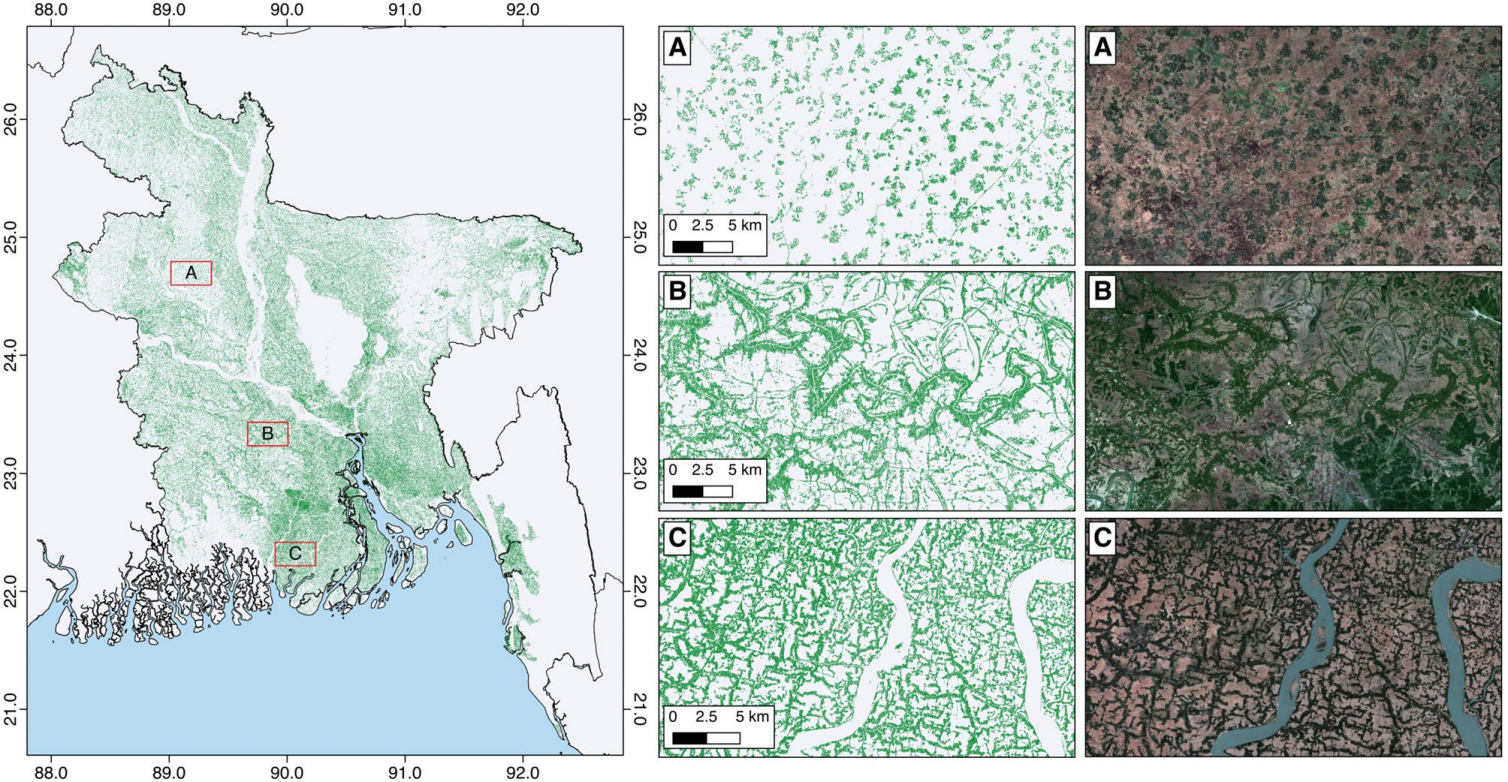
Distribution of trees outside forests (TOF) in Bangladesh. (**A**) Isolated stands of TOF in scattered dwellings (**B**) Less anthropogenically influenced TOF along rivers and geomorphological features (**C**) TOF in an agricultural region forming narrow stands around field boundaries. Map made with QGIS 2.8 (https://www.qgis.org/en/site/index.html).

TOF accounts for an additional 31% more tree area than existing estimates of TOF^22^ in Bangladesh. A total of 2,233,578 ha of TOF was mapped across the country (Fig. 1) accounting for 15.1% of the Bangladesh surface area. The area of TOF mapped is 53% larger than the existing mapped forest area of 1,464,000 ha^22^, highlighting the prominence of TOF in comparison to forest area in Bangladesh. The largest proportion of TOF was in the division of Dhaka (415,582 ha) with an almost equitable area of TOF in the division of Chittagong (399,331 ha). The area of TOF mapped per division is given in Table 1. Barisal contained the largest proportional TOF area (22.8%) while Sylhet contained the lowest proportional forest area (11.4%). TOF was mapped at the national scale with an accuracy of 91.5% (Supplementary Table S1), ranging from 89.3% in Dhaka to 93.7% Rangpur.

TOF within Bangladesh were characterized by numerous types of stands, varying in size and density across the full extent of the country (Fig. 1). In agricultural regions (Fig. 1C), the TOF were located along agricultural field boundaries and among nearby local settlements. Here the TOF occurred in narrow lines which connected at nodes to other stands of TOF. All settlements contained trees, particularly rural settlements with lesser tree extent mapped in large urban cities. Figure 1A demonstrates these small homestead trees that occur as isolated stands in geometric patterns that surround individual dwellings. These are not interconnected but occur as small single stands, separated by agricultural fields. Rivers were also commonly tree lined often accounting for the largest stands of TOF. Here the TOF were mapped as narrow stands, but as long tributaries connected to other similar TOF patches, forming large continuous connected extents at the landscape level. These had a larger width in relation to their length, defined as the largest axis of the TOF patch, than other stands of TOF. This is visible in Fig. 1B whereby the TOF occurs as narrow stands but in less geometric patterns across the landscape.

The majority (65.6%) of the TOF stands were below 1 ha in size, accounting for 9% of TOF area, with an additional one quarter (25.6%) below 5 ha (15.4% of TOF area). Almost all of the stands patches (99.7%) were below 100 ha in size, accounting for 62.3% of TOF area. TOF stands over 1000 ha (0.3% of stands) contained 37.7% of TOF area (Table 2). TOF is heavily fragmented in terms of the number of stands, but maintains connectedness as a small number of the patches constitute a large proportion of the TOF area. This connectedness is reflected in Fig. 1B and C where the stands remain connected at the landscape level despite being fragmented at the local level. These stands, while covering an area large enough to qualify as forest, remain classified as TOF due their setting among agricultural and urban land uses. Furthermore, these stands are connected by as little as a single pixel, thus do not necessarily have a continuous canopy. Examples of these patch sizes are provided in supplementary Figure S1.

**Table 1:** TOF area within the divisions of Bangladesh.

Administrative division	TOF (ha)	95% confidence interval (± ha)	Division area (ha)	% TOF
Barisal	303,220	25,123	1,329,700	22.8
Chittagong	399,331	37,392	3,377,100	11.8
Dhaka	415,544	29,102	2,059,300	20.2
Khulna	293,262	37,441	2,227,200	13.2
Mymensingh	178,410	15,544	1,058,400	16.9
Rajshahi	267,266	36,763	1,819,700	14.7
Rangpur	233,180	28,632	1,631,700	14.3
Sylhet	143,365	19,365	1,259,600	11.4
Total	2,233,578	69,819	14,812,700	15.1

Associated with each division is the 95% confidence interval on the mapped area and the total proportion of area that TOF occupies in each division.

**Table 2:** The number and size of TOF stands in Bangladesh and the % of stands and % TOF that they account for.

Connected area (ha)	Number of patches	% of patches	% TOF
$\le$ 1 ha	454,846	65.6	9
$\ge$ 1 $\le$ 5 ha	177,909	25.6	15.4
$\ge$ 5 $\le$ 10 ha	29,928	4.3	8.5
$\ge$ 10 $\le$ 100 ha	29,154	4.2	29.6
$\ge$ 100 $\le$ 1000 ha	1904	0.3	17.1
$\ge$ 1000 $\le$ 10,000 ha	119	< 0.1	12.4
$\ge$ 10,000 $\le$ 100,000 ha	8	< 0.1	7.9

### Comparison with existing forest maps

**Figure 2:**
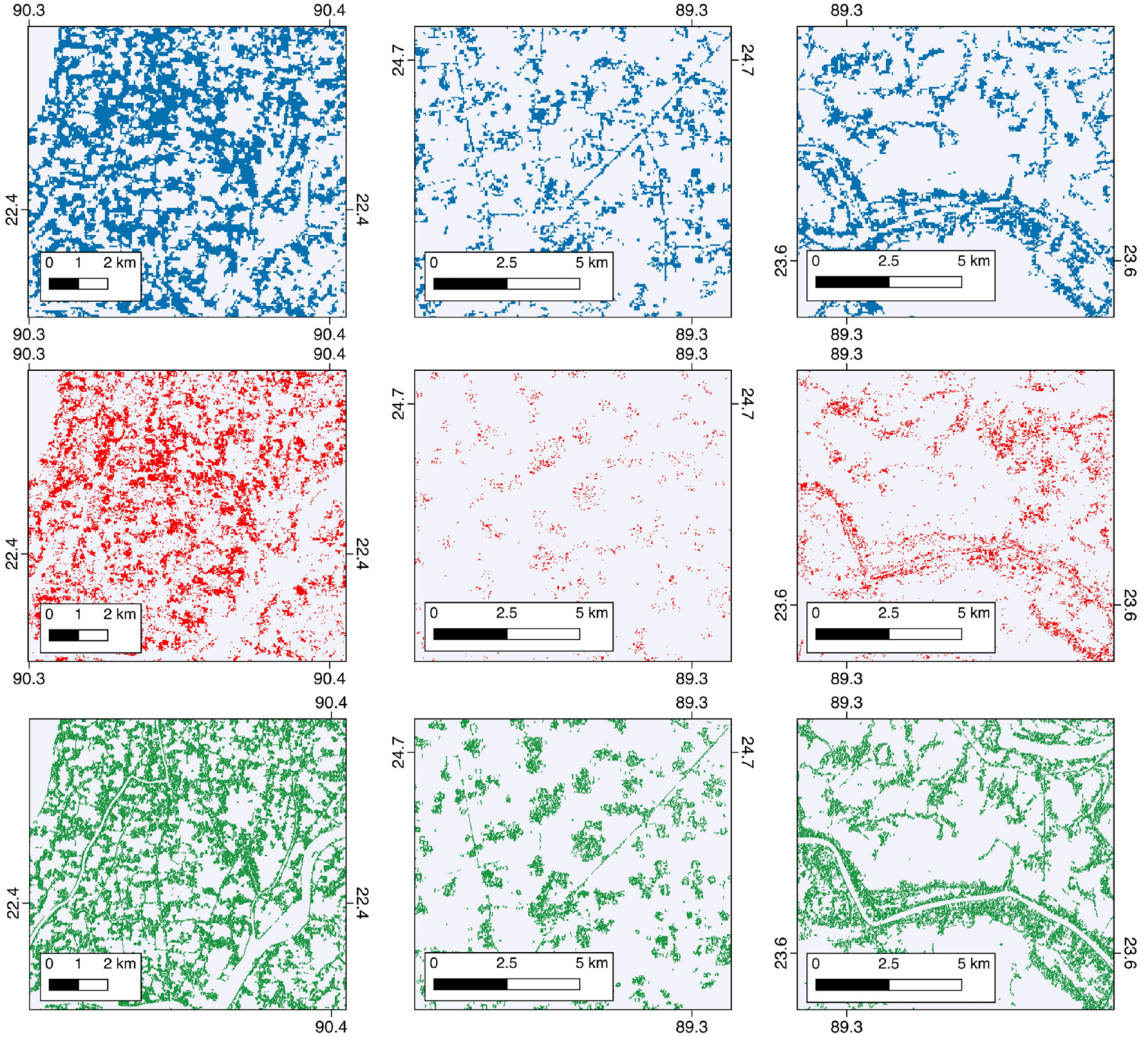
A comparison between the DLR FNF map (Top), extent-equivalent modified Hansen^32^ map (Middle) and TOF map (Bottom). Spatial resolution drives the differences between the maps, with most detail captured by the TOF map. The TOF and DLR FNF map^33^ are most similar, perhaps due to the use of small wavelength radar while the global forest cover map does not capture small forest stands. The differences and similarities between the maps reflect the modes of data used and the metric that was mapped (cover or extent). Maps made with QGIS 2.8 (https://www.qgis.org/en/site/index.html).

Our Sentinel-derived TOF map estimates a greater quantity of tree extent across every division in Bangladesh than the comparable global forest cover map of Hansen et al.^32^ when converted from canopy cover to extent (see methods). The largest difference between the maps occurred in Dhaka where 286,862 ha more were mapped at the 10 m scale. The smallest discrepancy was 38,939 ha in Barisal which exemplifies the large differences in tree extent mapped at these two different scales. The TOF map presented is of all vegetation of a height greater than 2.19 m, thus we applied a 5m height threshold as used by the global forest cover dataset^32^. Using this threshold we map a greater area of TOF in all divisions, with the exception of Sylhet. The area per division in each map is given in Table 3, alongside the area of TOF greater than 5 m. Similarly, the TOF map was compared with the 50 m German Aerospace Agency (DLR) Forest/Non-Forest (FNF) map^33^ which mapped a greater quantity of forest in five of the eight divisions. The largest difference occurred in Khulna where 123,028 ha more forest was mapped in the FNF dataset, although small differences were mapped in Rajshai and Sylhet where the FNF quantified 20,956 ha and 29,380 ha less, respectively. The differences between these datasets is given in Fig. 2, which shows the discrepancies and similarities between the three maps. Our TOF map resolves stands in the FNF map in greater detail and includes TOF largely omitted from the global tree cover map. Our estimate of TOF extent is 532,178 ha (31.3%) greater than the previous remotely sensed estimate of TOF forest cover area by Potapov^22^, although restrictions on the distribution of this dataset prevent a more thorough quantitative analysis.

**Table 3:** Area of TOF mapped compared with that of a canopy height greater than 5 m and in comparison with the extent-equivalent modified Hansen^32^ dataset and DLR FNF map^33^.

Administrative division	Area of TOF (ha)	Area > 5 m (ha)	Hansen et al. area (ha)	TDX FNF area (ha)
Barisal	303,220	213,075	158,773	331,797
Chittagong	399,331	277,181	224,110	463,168
Dhaka	415,544	265,083	128,682	327,624
Khulna	293,262	194,372	149,802	419,290
Mymensingh	178,410	143,529	73,514	202,110
Rajshahi	267,266	183,087	51,583	246,310
Rangpur	233,180	134,708	61,654	362,804
Sylhet	143,365	66,532	104,426	113,985

### Canopy height

**Figure 3:**
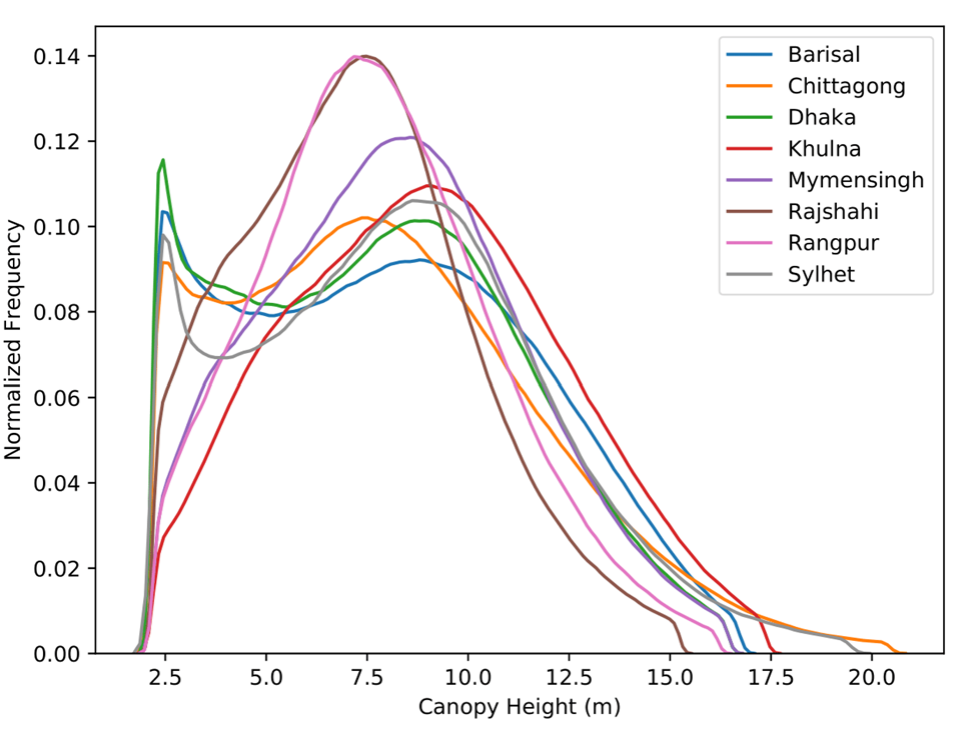
Distribution of TOF height for each division in Bangladesh. Minimum heights are 2.19 m and maximum heights are limited to the 99th percentile.

**Figure 4:**
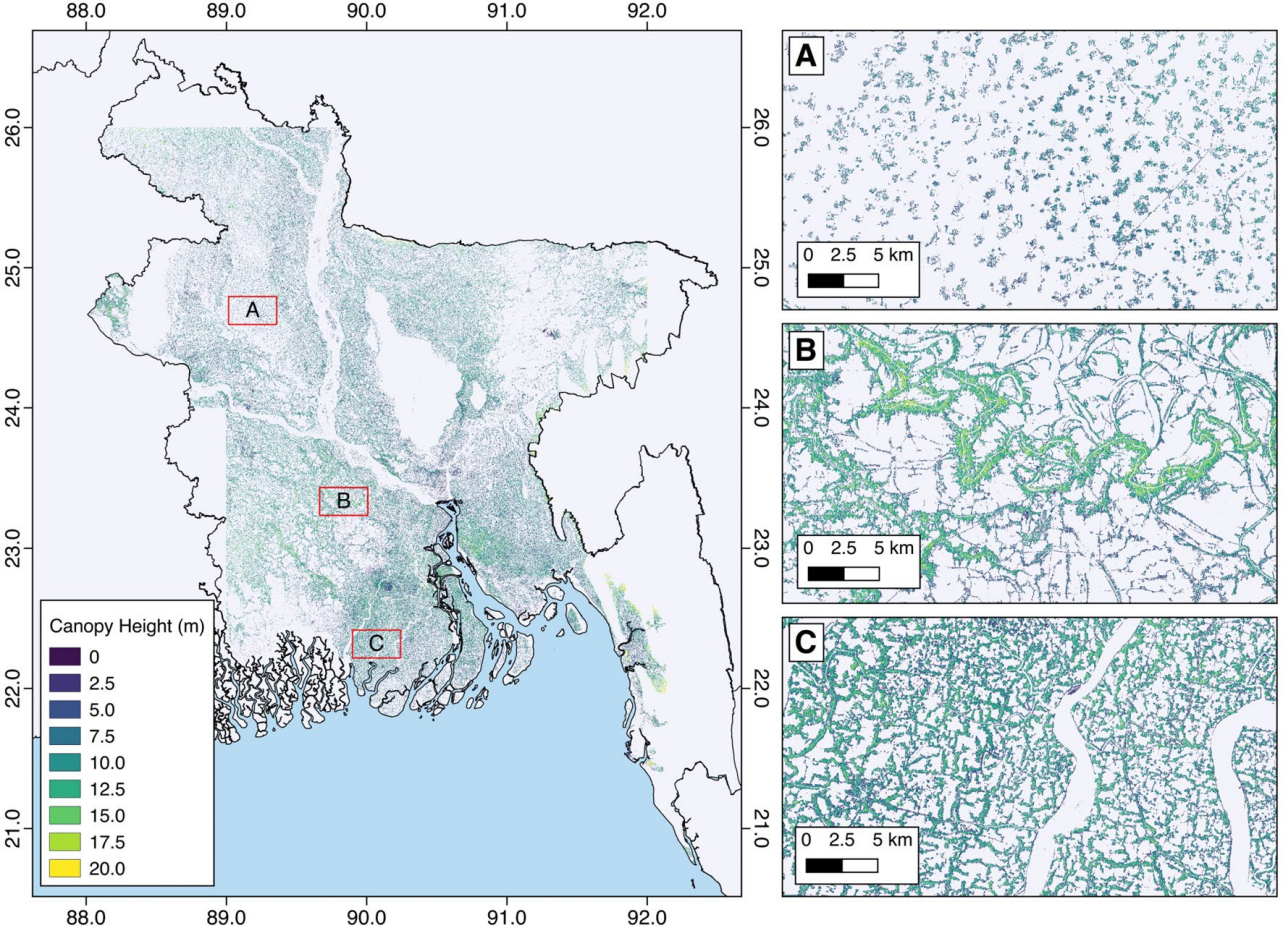
Distribution of TOF heights in Bangladesh. (**A**) Small heights surrounding homestead farms and dwellings (**B**) Larger TOF heights in natural stands along rivers. Larger trees are at the center of the stand and decrease towards the stand margin (**C**) Tree heights in anthropogenically managed TOF stands that line field boundaries. TOF stands on elevated terrain were omitted, alongside continuous forests and mangroves. Map made with QGIS 2.8 (https://www.qgis.org/en/site/index.html).

TOF canopy height was generated at the national scale at a resolution of 30 m for Bangladesh. The average height of TOF was 7.3 m. The division of Mymensingh contained the tallest average TOF height at 8.2 m and Sylhet contained the shortest average TOF height of 6.0 m. The average and maximum TOF heights are given in Table 4. The Chittagong contained the tallest maximum canopy height stand at 20.5 m while the shortest maximum canopy height tree stand was in Rajshahi (15.2 m). The distributions of the tree heights by division is given in (Fig. 3). There was no relationship between height and spatial distribution at the national scale with a range of heights observed across the landscape, although localized relationships were more evident. This was more prominent at the stand level, whereby larger stands contained taller trees, with smaller trees at the outer edge of the stand, increasing in height towards the stand center (Fig. 4C). TOF that were isolated within homestead settings contained shorter trees (Fig. 4A) while TOF that were more continuous (Fig. 4B) contained taller trees. Canopy heights varied across the landscape with adjacent pixels often displaying a contrast in heights, indicative of heterogeneous canopies. The average tree height increased when only trees above 5 m were accounted for, as expected when the large number of small trees were removed. These average tree canopy heights above 5 m are also provided in Table 4.

**Table 4:** Average and maximum TOF heights per division in Bangladesh, with average tree height for trees above 5 m only.

Administrative division	Average height (m)	Max height (m)	Average height (TOF >5 m)
Barisal	8.1	16.7	9.6
Chittagong	8.1	20.5	9.5
Dhaka	7.8	16.4	9.3
Khulna	9.0	17.4	9.8
Mymensingh	8.2	16.4	9.2
Rajshahi	7.3	15.2	8.3
Rangpur	7.8	16.2	8.6
Sylhet	8.3	18.5	9.6

## Discussion

We provide among the first country scale canopy height maps^34^ and the first TOF canopy height map for Bangladesh, a country with a highly fragmented and distributed wood resource. At 10 m spatial resolution, we provide the most recent and detailed map of TOF extent for Bangladesh alongside associated canopy height at a resolution of 30 m.

Our map of TOF increases previous estimates by 328,541 ha (31.3%) over that of Potapov^22^, 816,941 (67.3%) over that of the FAO FRA^35^ and 1,281,034 ha (57.4%) over the global forest cover map^32^. The DLR FNF^33^ estimate was 10% larger than our mapped extent, which was the smallest discrepancy from our TOF map. This small difference may reflect the use of radar imagery, which is sensitive to tree structure, used in both methods and the coarser pixel resolution whereby an increase in backscatter from a small stand could be the dominant scatterer even at 50 m resolution. There was a larger difference with the other approaches that used optical remotely sensed imagery alone, but these estimates were closer to our TOF extent than the FRA estimate. While different methods and definitions of forest were used in the creation of each map and should be considered when comparing these datasets, this also highlights that although definitions are important for broadly classifying forests, they are a dynamic and continuous factor. The consequence of this is a risk of excluding large quantities of trees, particularly where TOF is a large proportion of a nations wood resource.

The influence of anthropogenic activity upon the forests of Bangladesh and the rise of agriculture which replaced it^36^ was reflected in the area of TOF within the eight divisions of Bangladesh. The large area of TOF in each division reflects the widespread loss of forest and conversion to agriculture in Bangladesh, particularly outside of elevated regions, leaving large quantities of TOF remaining. The total area of TOF was equitable to 0.014 ha per person, in contrast to the US where the FAO definition of Other Land with Tree Cover (OLwTC) equates to 0.08 ha of forest per person. This requires TOF in Bangladesh to provide ecosystem services to a larger number of people from a smaller area covered by trees. As TOF were the only trees in some divisions, this provides the only resource available to local populations. These TOF were observed to consist of many types which the Bangladesh government has promoted^20^ through agroforesty, homegardens, farm forestry, social forestry and urban forestry, among others^37^. Some of these are identifiable in the tree extent map, particularly those of the homegardens and the urban forestry. These TOF are critical as they are able to serve as proxies for forest, allowing the rural and landless poor to procure subsistence products such as fuel wood, fodder and timber. In this way, TOF contribute substantially to the rural economy through the provision of products which reduces the reliance on purchasing power, while also offering employment^38,39^. In fact, economic drivers have been primarily responsible for the documented increase of TOF in Bangladesh although landowners are also able to appreciate the secondary environmental benefits that TOF provide, which can often be entwined with economic factors^40^. The variation in the stands that TOF form reflects the range of ways in which TOF is utilized and subsequently manifests as a heterogeneous TOF type at the national level.

There was a large variation in TOF stand size, which was a function of the relationship between the size of the stands and their connectedness. Over 90% of the stands were <5 ha in size, accounting for 24.4% of total TOF, reflecting the disparate nature of TOF, promoted by homestead and social forestry that are not well connected and occur as isolated patches. However, the majority of the TOF area (76%) occurred in stands greater than 5 ha when stands joined by at least one pixel were mapped, which suggests a connectedness between remaining TOF despite heavy anthropogenic management. Their location in agricultural and urban settings qualify these stands as TOF but this connectedness allows ecological continuities between forest patches, over large fragmented stands at the landscape and national level^41^. Bangladesh TOF is host to significant species diversity, particularly in insect, avian and tree species despite the amount of area it occupies^42^, thus the connectedness of TOF within Bangladesh serves an important ecological function despite accounting for 15.1% of the land area. Additional losses would have a disproportionately negative impact as the remaining TOF serves an ever important ecosystem function. As the area demarcated for natural forest in Bangladesh is low and almost all limited to specific locations, TOF is a keystone in ensuring ecological richness and diversity^40^ across the country. The ability of TOF in Bangladesh to meet these demands, is not guaranteed into the future without the continuation of the commitment of local level parties and grassroots movements to promote participatory social forestry and increase TOF^16,18^.

The disparity between our TOF map and other mapped extents is a consequence of two controlling factors. The first is definitions of forest and TOF which can be susceptible to interpretation and thus affect the categorization of tree type. We interpret the majority of trees within the Bangladesh deltaic plain as TOF due to the heavy presence of agricultural and urban land uses, which have fragmented the remaining woody resource. We therefore align our definition of TOF with that of the FAO^12^ definition of Other Land with Tree Cover. In contrast, the Potatpov^22^ map uses a combination of tree cover (>50%) and user assigned labels within a sampling approach to classify forest type. The Hansen^32^ map used in our comparison includes all pixels with >0% canopy cover and the DLR FNF map is a binary tree map with no categorization into forest type^33^. Each map is justified in its choice of definition but highlights that maps are not reliably comparable due to an lack of consistent definitions and the comparison of forest with non-forest pixels. Secondly, we use a high spatial resolution of 10 m, increasing the resolution over other remotely sensed methods^33^ by as much as 25 times. This enables the detection of small stands that are below that of coarser resolution imagery, but which are common in TOF. This combined with differences in definitions drive discrepancies between mapped products and while we report different metrics than existing maps, we do not claim a holistic superiority over them.

The average tree height varies by 1.7 m between divisions although the distribution of the TOF heights exhibit marked differences. Maximum tree heights differed by 5.3 m and the divisions of Barisal, Dhaka and Sylhet had an increased quantity of small trees compared to the other divisions. This variation may reflect the different ways in which TOF are used across the country and the difference between rural, urbanized and agricultural regions, whereby tree stands may be affected by use intensity or allowed to grow in order to provide other services such as shade^43^. TOF have been observed to be used as wind breaks and can be grown in mixed stands which form a semi-permenable barrier over a range of heights. This may drive the variation in tree heights as part of efforts to reduce soil erosion and desertification in more arid regions^37^. Average tree height increased within each division when a 5 m minimum threshold was used, as is stated within FAO FRA guidelines for forest. This was accompanied by a reduction in TOF area, suggesting that much of the TOF is below 5 m in stature. This reflects the heavy dependence of the population on their TOF resource, as shorter woody vegetation emerge in the absence of forestry. The 5 m threshold aligns this work with previous estimates^35^ but results in the underestimation of trees which may be responsible for the large discrepancies between the TOF map and existing datasets. Therefore, a 5 m threshold may not be suitable for accounting for woody resources which can be mapped with high-resolution (10 m) imagery and should be reconsidered with the emergence of high resolution extent mapping. The temporal difference between our tree map (2018) and elevation data (2011–2013) is a potential source of error, as tree extent undoubtedly changed during this period. However, between 2000–2014 an overall tree gain of 4.3% was observed across Bangladesh^22^, thus we do not expect this to be a substantial source of error. Furthermore, the use of averaged tree heights in 1 ha plots, as outlined in the methods, was designed to reduce the impact of anomalous values on our tree height map. To derive a tree height map, a bare earth surface model was generated to subtract from the TDX DEM. We provide a labor intensive but accurate method of creating bare earth elevation models in regions with modest changes in elevation and slope. This method is applicable to other regions, particularly coastal zones and agricultural areas which are often characterized by small changes in elevation and sufficient locations from which to choose ground control points (GCPs). This provides a means of generating digital surface models (DSMs) from openly available elevation data such as SRTM, for vegetation height mapping across broad geographical scales.

Given the often disparate distribution of TOF, maps of extent and canopy height can provide information for resource accounting at the national scale once combined with field collected data. Field surveys are time intensive and expensive, therefore remote sensing provides a means of directly measuring extent and structure across landscapes. Our maps are directly applicable for use in tandem with a Forest Resource Assessment (FRA) or National Forest Inventory (NFI) by capturing the spatially explicit distribution of TOF and reducing the uncertainty in these smaller reporting units where high fragmentation and inaccessibility can provide barriers to comprehensive reporting^2^. This would increase the capability for resource monitoring, particularly where TOF are the only wood resource. Prior to this, there was no knowledge on the structure of TOF which extends across almost the entirety of the Bangladesh. Knowledge on the distribution of height will be pertinent to maximizing efforts to manage this living biomass and the subsequent distribution of the ecosystem services that it can provide. This has important environmental and economic implications in terms of carbon storage and timber, respectively, and provides additional uses at the local level^37–39,43^. We envisage that our height map may be utilized as an input into national biomass accounting, when combined with additional field data, which is relevant to carbon accrediting schemes such as REDD+. Given the large proportion of tree area composed of TOF, this equates to a large percentage of the national carbon budget within living biomass, yet the proportion of carbon fluxes from changes in TOF extent are not yet known. This may overlook the importance of the inclusion of TOF and sparse forest types in national accounting and emissions estimates, even when they compose significant proportions of aboveground biomass. Our maps can help ensure this and with the recent availability of tree height estimates from spaceborne LiDAR missions (e.g. GEDI, ICESat-2), we anticipate that TOF canopy height will become commonplace when combined with accurate estimates of tree extent.

Given the fragmented distribution of TOF, spaceborne sensors with increased spatial resolution will be instrumental in resource monitoring of TOF. The 10 m pixel resolution facilitated the mapping of small stands of trees, over openly available data traditionally used for large scale land cover mapping (e.g., Landsat)^32^. The Sentinel-1 imagery was an ideal companion to Sentinel-2 as the combination of the radar imagery, indicative of structure, with the optical imagery for estimating land cover “greenness”, provided a novel partnership for land cover mapping that has not previously been witnessed as part of the same satellite constellation. The availability of sufficiently high-resolution high-cadence imagery, particularly when accessible as analysis-ready-data in cloud computing environments (e.g., Google Earth Engine), position them as a solution to prior limitations of routine TOF mapping^2^.

TOF accounts for 31% and 52% more TOF and forest, respectively, than presently estimated^22^ in Bangladesh, accounting for a significant proportion of the woody resource. This discepancy is driven largely by differences in the definition of TOF and the higher resolution dataset used in this study. We demonstrate that this information can now be readily supplied for resource accounting through the use of openly available high-resolution spaceborne imagery and easily accessible cloud computing environments. Furthermore, we demonstrate a method for achieving canopy height estimates from existing elevation data, which can be used in combination with TOF maps to provide comprehensive data on TOF extent and structure to inform national forest inventories/resource assessments at the national scale. We highlight that in countries such as Bangladesh, a focus on forest definitions detracts from achieving the most informative products for resource accounting, particularly for those that require management at fine scales in order to most effectively benefit populations at the local level.

## Methods

### Study site and datasets

#### Bangladesh

**Figure 5:**
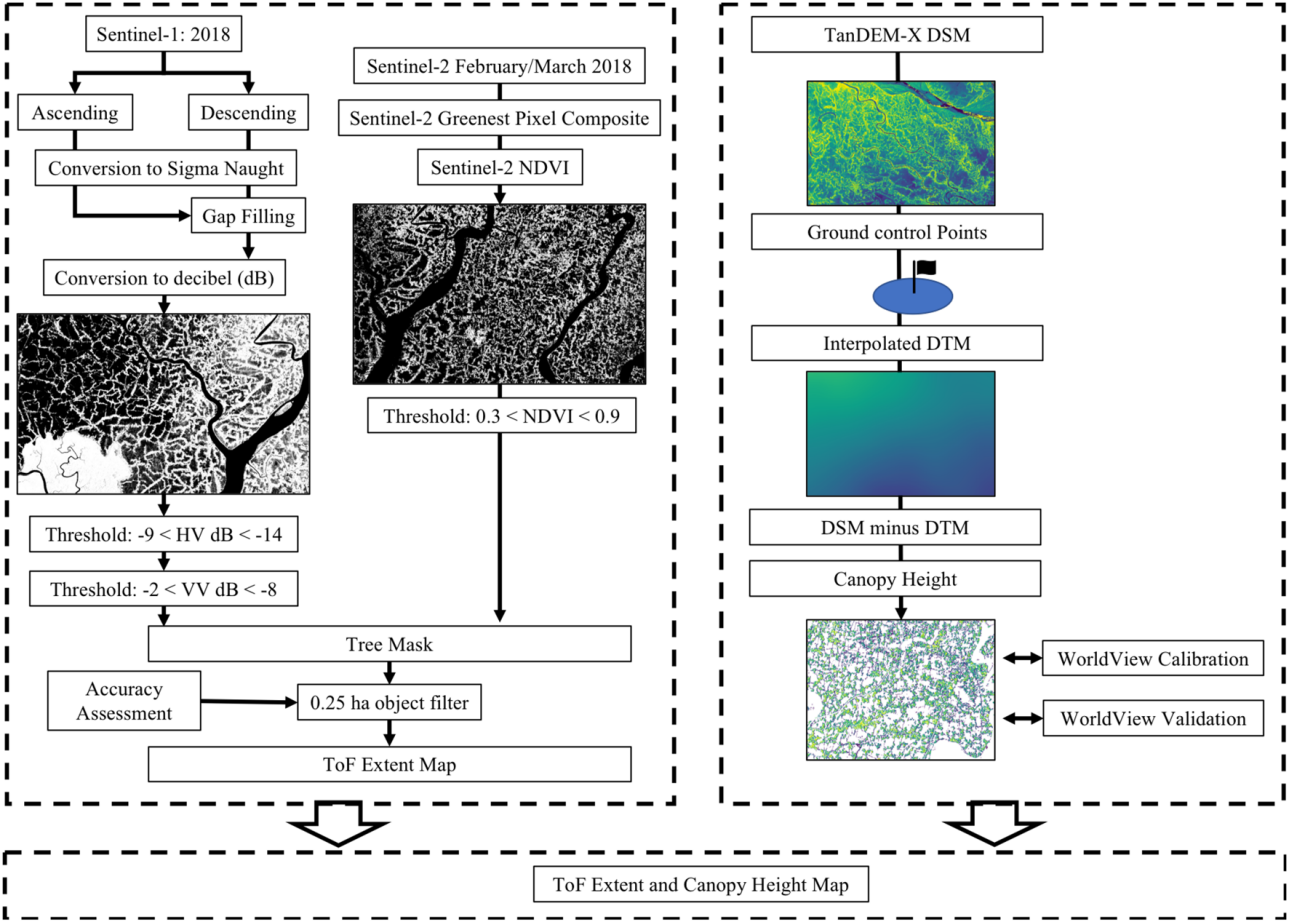
An overview of the datasets and steps used to generate a high-resolution tree extant and canopy height model for TOF in Bangladesh.

Bangladesh is a South Asian country located between 21$^\circ$ - 27$^\circ$ N and 88$^\circ$ - 93$^\circ$ E, with an administrative area of 147,570 km^2^ and a population of 161,356,039^44^. Bangladesh is geographically divided into three regions of the Ganges-Brahmaputra delta and the Madhupur and Barind plateaus. Evergreen hill ranges make up the northeastern and southeastern portions of the country but the majority of Bangladesh is flat low-lying land with an average elevation of 12 m above mean sea level, at the exception of the southeastern and northeastern hill tracts that increase to approximately 2000 m. Bangladesh has a tropical climate with warm and humid summers (March–June) and mild winters (October–March) with monsoon season from June to October. The vegetation of Bangladesh are composed of tropical moist deciduous and semi-evergreen forests, freshwater wetlands and mangrove forests^45^. The country is administratively divided into 8 divisions: Barisal, Chittagong, Dhaka, Khulna, Mymensingh, Rajshai, Rangpur and Sylhet. An overview of the method for mapping TOF extent is given below and in Fig. 5.

#### Sentinel-1

Sentinel-1 operates as a pair of satellites composed of Sentinel-1A and Sentinel-1B. Each is a C-band Synthetic Aperture Radar (SAR) collecting dual polarization images of Earth’s surface, with a repeat orbit every 6 days at a spatial resolution (pixel size) of 10 m. Radar instruments, such as those aboard Sentinel-1, are not inhibited by cloud cover nor atmospheric conditions, enabling data to be collected consistently over tropical regions during both day and night. Radar instruments are active sensors and transmit a pulse of energy towards Earth’s surface and measure the response, termed backscatter. The quantity of backscatter returned to the sensor is a function of the target properties such as shape, size and water content and a function of the transmitted wavelength. Large, rough and sensor facing targets return a greater quantity of backscatter than small or smooth targets or those oriented away from the sensor. Sentinel-1 imagery is available within Google’s Earth Engine environment where it is radiometrically calibrated, terrain corrected and in log scale (dB) units.

#### Sentinel-2

Sentinel-2 is a satellite constellation composed of Sentinel-2A and Sentinel-2B. Each carries a passive multispectral instrument with 13 visible, near-infrared and shortwave infrared bands with spatial resolutions of 10, 20 and 60 m. Sentinel-2 acquires images over the same location on Earth’s surface every 5 days in high latitudes, with a field of view of 290 km. The range of multispectral bands measure the quantity of light that is absorbed or reflected from different land cover types. Sentinel-2 imagery can therefore relay information on the water content, photosynthetic cover and bio-physical properties of land cover types. As with Sentinel-1, Sentinel-2 imagery is available within Google’s Earth Engine environment.

#### TanDEM-X DEM

TerraSAR-X is an Earth observing x-band SAR instrument operated by the German Aerospcace Agency (DLR), launched in 2007. Since 2010 TerraSAR-X was accompanied by an almost identical second satellite. The two satellites fly in a helix formation, enabling the generation of interferograms with no temporal decorrelation and adverse impacts from atmospheric disturbances. The interferogram enables a digital elevation model (DEM) to be generated with a spatial resolution of 12 m and a relative vertical accuracy of 2 m. TanDEM-X is available from DLR through a science proposal.

### Mapping tree extent

The mapping of tree extent was carried out in Google’s Earth Engine environment, allowing instant access to imagery and algorithms to create cloud free optical composites and radiometrically calibrated and terrain corrected radar mosaics. To minimize cloud cover over Bangladesh, Sentinel-2 optical scenes were acquired during the dry season between February 1st 2018 and March 31st 2018, utilizing 824 Sentinel-2 10,000 km$^2$ tiles. For each image in the collection, the NDVI was calculated. This was used within the Earth Engine Quality Mosaic function to create a ‘greenest' image composite, whereby a single image is generated using the pixel location of the most photosynthetic pixel from each image in the collection. From this composite, a single NDVI image was generated to determine photosynthetic pixels whilst avoiding differences in atmospheric conditions and subsequent reflectance. Thresholds on NDVI were used to segregate photosynthetic from non-photosynthetic land cover using manually defined thresholds (0.3< NDVI<0.9). The result of this was a binary map of photosynthetic land cover. Thresholds were chosen with constant reference to the imagery and underlying VHR Google Earth imagery which is readily achieved using the GEE interface.

Sentinel-1 imagery was acquired for the whole of 2018, from January 1st to December 31st, totaling 771 images from both ascending and descending passes. Although the radar is not inhibited by illumination nor atmospheric conditions the data can be degraded by image speckle. To reduce this the imagery was averaged over a large temporal range to improve the quality of the imagery, based upon the assumption that trees in Bangladesh are a static land cover type relative to other land cover types (e.g., agriculture) and the backscatter will remain consistent. Both ascending and descending scenes were acquired forming two collections. Both the VH and VV polarized bands were selected from interferometric wide (IW) swath mode imagery. Separate ascending and descending images were generated by averaging the images in each collection. Prior to averaging the imagery was converted to Sigma Nought from the Earth Engine provided decibel (dB) units. Scan lines of very low backscatter between swaths in the average descending image were filled with values from the average ascending image. Each polarization was converted to decibel (dB) yielding two backscatter images. Manually defined VH backscatter thresholds of −9 to −14 dB, were used to segregate land cover with structure from those without. The VH polarization is indicative of volumetric scattering, such as that from a tree canopy, which denotes structure. The VV polarization was used to reduce false positives by removing pixels that were outside of the backscatter thresholds from −2 to −8 dB. Thresholds were chosen with constant reference to the radar imagery and underlying VHR Google Earth imagery within the GEE interface.

The intersection of the radar and optical binary maps combined pixels identified as photosynthetic with pixels identified as having structure, synonymous with trees. The use of decision thresholds were preferred over supervised classification algorithms. As atmospherically corrected data were not available, only normalized optical image indices in combination with two radar bands could be used, which limited the data layers available. Also, given the large geographical area, a large but unknown number of classes would need to be trained, increasing potential misclassification. Our approach therefore is simplified yet robust based upon our accuracy assessment and is in line with other radar derived remote sensing derived forest maps^46^. Adjoining pixels were clumped into objects and those composed of less than 25 pixels (0.25 ha) were omitted, in order to reduce the detection of small false positives and noise. The combination of these two sensors yielded a high resolution country wide map of all trees in Bangladesh with a combined crown cover greater than 0.25 ha. TOF were separated from forests using a manually drawn mask with reference to the land use in very high resolution Google Earth imagery. These forested regions were characterized by their setting outside of agricultural/urban settings and with large continuous canopy cover. The land area omitted is visible in supplementary Figures S2 and S5. The thresholds chosen were considered to be conservative, in order to reduce the false classification of trees in the radar imagery. This reduced the noise in our data but also led to the underclassification of some pixels. This was most common among more continuous stands of TOF whereby heterogeneous surfaces caused destructive interference and the reduction of backscatter in radar imagery. Plantations were not readily separable from other forests in the imagery and were considered a form of TOF. Patch size was determined by clumping TOF pixels that were connected by at least one pixel edge and calculating the area of each.

### Digital terrain and canopy height models

TanDEM-X (TDX) elevation data was acquired 2011–2013 and covers almost the entirety of Bangladesh. In order to attain the extent required, the highest resolution (12 m) data was supplemented with coarser resolution (30 m) data to maintain wall to wall consistency. The TDX measures surface height so it was not possible to directly measure canopy height from the ground surface as the terrain elevation was unknown. Areas of bare ground were sampled with 8200 points, using the TDX and Google Earth imagery as reference. A Geospatial Data Abstraction Library (GDAL^47^) inverse power to distance interpolation was used to estimate the ground elevation and a sample of 400 independent points over bare surfaces were used to calculate an RMSE of 1.2 m. A map of the 8200 GCPs and 400 validation points is given in supplementary Figure S4 alongside a contour interval map of the interpolated surface in supplementary Figure S5. The interpolated surface was subtracted from the TDX DEM and intersected with the tree extent map to generate a canopy height map for the extent of the TDX imagery. Areas of continuous forest and elevated terrain were omitted as closed canopies prevented the accurate extraction of ground points and the interpolated surface could not be adequately fit in areas of rapidly changing slope. These regions contained predominantly continuous forests and not TOF. The DTM is provided in supplementary Figure S2.

### Canopy height model calibration

**Figure 6:**
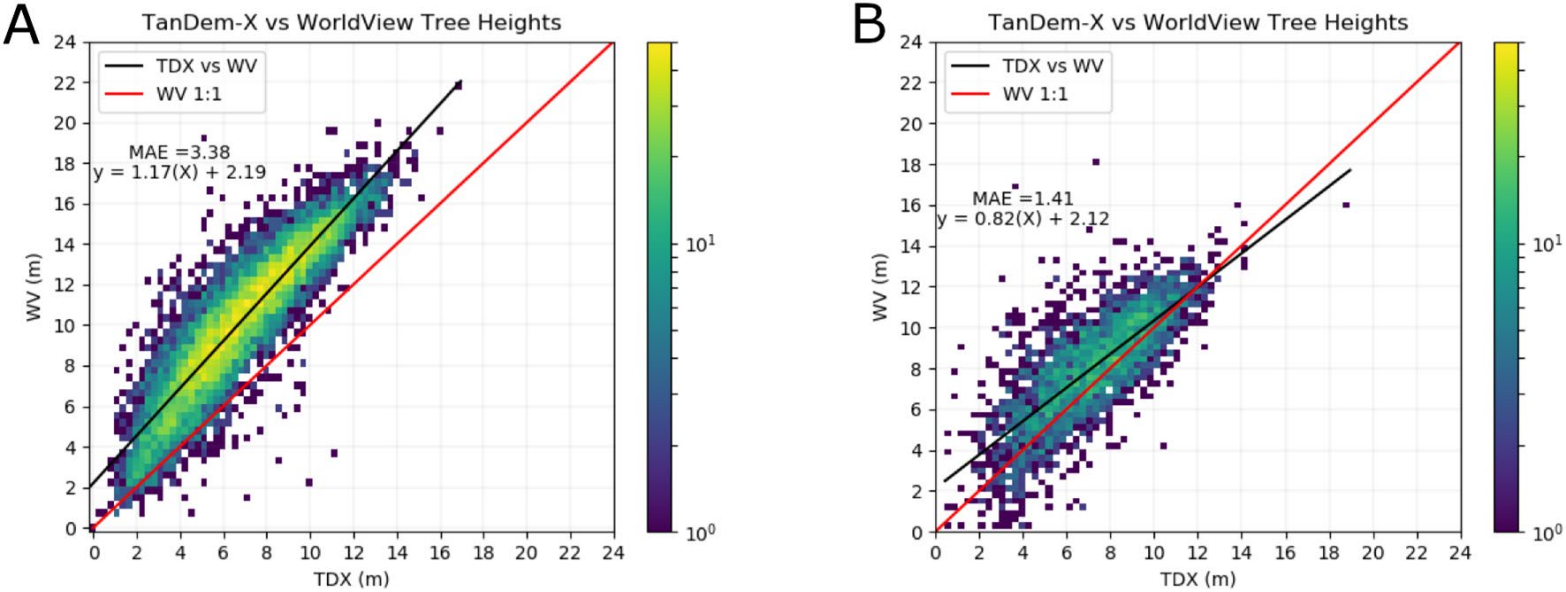
Calibration and Validation of the TanDEM-X derived canopy height estimate, with reference to WorldView Stereo Imagery. (**A**) The calibration of the canopy height map. (**B**) A correction from (**A**) was applied and the canopy height was compared to an independent WorldView Stereo Image.

The canopy height model was dependent upon the accuracy of the TDX DEM and the interpolated surface. Derived from radar, the elevation value is dependent upon the penetration of the radar wavelength into the canopy and was susceptible to changes with natural variations in the physical structure of the canopy. To calibrate and validate this, the TDX canopy height model (CHM) was compared to canopy surfaces captured in digital surface models (DSMs) derived from high resolution spaceborne imagery (HRSI). These HRSI DSMs were derived from stereoscopic image pairs collected during along-track acquisitions by the Worldview-2 satellite. This imagery has been demonstrated to successfully model canopy surfaces and derive canopy heights comparable with lidar^48–50^. WorldView stereo imagery was divided into calibration and validation datasets, using a 2013 dataset for calibration and a 2016 dataset for validation. The TDX canopy height model was subset to the WorldView calibration extent and both datasets were reprojected and resampled to 10 m and the TOF tree extent map masked out the ground values in both DEMs. The average elevation value from the TDX CHM and WorldView stereo imagery was extracted using 9499 1 ha plots. A 1 ha plot was chosen to reduce errors associated with geolocation or classification error. The relationship between the two elevation datasets assessed the performance of the TDX CHM (MAE: 3.38 m) and provided coefficients to improve the TDX CHM (Fig. 6A). This was applied to the canopy height model and the process was repeated with an independent WorldView Stereo image and 3024 1 ha plots, yielding a new relationship (Fig. 6B) and reduced MAE of 1.41 m. The validation dataset contained a small number (12) of erroneous values that were greater than twice the 99th percentile of the difference between the DEMs and were excluded. We set a minimum tree height> 2.19 m as derived from the canopy height calibration model, removing any erroneously classified TOF pixels at or below 0 m prior to CHM calibration, thus we confidently measure tree height above 2.19 m. The maximum tree height was limited to the 99th percentile for each division, in order to eliminate erroneous canopy heights. A map of the location of the 1 ha calibration and validation points are available in supplementary Figure S3.

### Comparison with existing maps

Existing high-resolution maps of forest extent in Bangladesh were not available and the Potapov et al.^22^ map was not openly available. One of the most widely used tree cover maps is that of Hansen et al.^32^, that maps global tree cover at 30 m using Landsat data. This dataset maps tree cover per 30 m pixel and is therefore not a direct comparison with the extent map presented here. In order to make these maps more comparable, the tree cover map was converted to a binary mask where all tree cover pixels over 0% were converted to binary tree pixels. The Hansen et al.^32^ year 2018 baseline was combined with all gain pixels for Bangladesh up to the year 2012 to generate a maximum tree extent map for the period. Similarly, the DLR TanDEM-X Forest/Non-Forest map^33^ was downloaded from DLR and was also compared with our TOF map. This dataset uses TanDEM-X interferometric SAR (InSAR) to classify forests globally at 50 m. This map, although at a coarser resolution to the global forest cover dataset, generates a binary map of forest extent that can be compared to our TOF map. Both datasets were either already available or uploaded to the Google Earth Engine, masked to match the TOF bounds and the area of forest in each division was calculated.

### TOF accuracy assessment

The accuracy of the TOF map was assessed with reference to Microsoft Bing Airborne Imagery, available in QGIS. A custom plugin tool was used to manually verify 500 validation points for two classes (TOF/Non-TOF) per division. This created a total of 8000 manually validated points. Each point was buffered by 10 m to account for possible geolocation error or differences in the resolution of the TOF map and reference imagery. The 95% confidence interval on the area of each division was calculated following suggested best practices^51^. All points were combined to calculate the overall accuracy of the TOF map at the national level. The national confusion matrix is available in supplementary Table 1.

## Supplementary Information

Supplementary Information.
