# Supplementary material for: Trees outside forests are an underestimated resource in a country with low forest cover
Source: Sci Rep. 2021 Apr 12;11:7919. doi: 10.1038/s41598-021-86944-2 (PMC8041888; doi:10.1038/s41598-021-86944-2)
Supplement: Supplementary file 1 — Supplementary Information. [file 41598_2021_86944_MOESM1_ESM.docx]

Trees Outside Forests are an underestimated resource in a country with low forest cover

Nathan Thomas1,2,*, Priscilla Baltezar1,2, David Lagomasino3, Atticus Stovall2, Zaheer Iqbal4, and Lola Fatoyinbo2

1Earth System Science Interdisciplinary Center, University of Maryland, College Park, MD 20740, USA

2NASA Goddard Space Flight Center, Biospheric Sciences Laboratory, Greenbelt, MD 20771, USA
3Department of Coastal Studies, East Carolina University, Wanchese, NC 27981, USA

4Resource Information Management System (RIMS), Bangladesh Forest Department, Dhaka 1207, Bangladesh

*nathan.m.thomas@nasa.gov


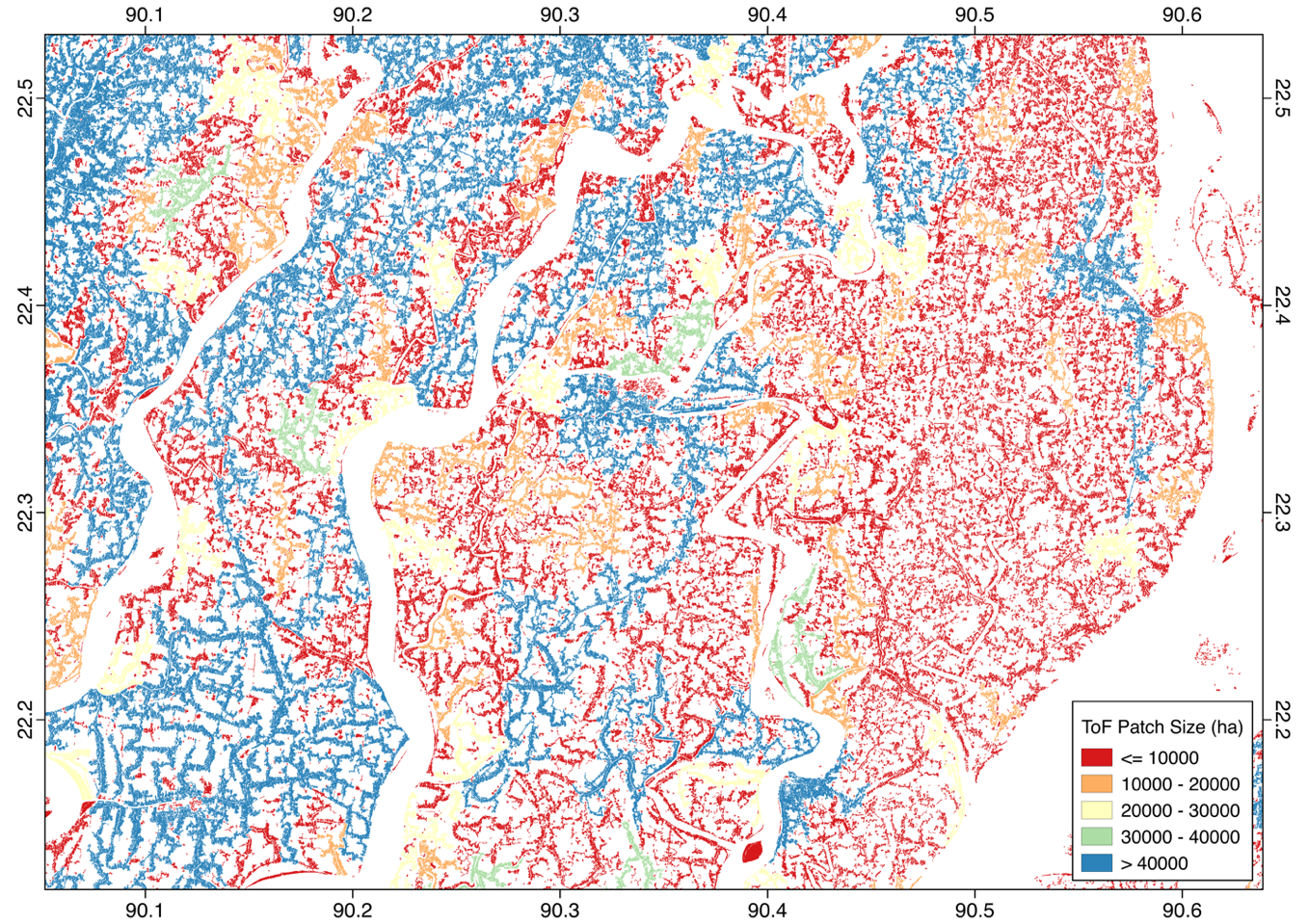


Figure S1. Examples of the patch sizes between stands connected by at least one pixel. Map made with QGIS 2.8 (https://www.qgis.org/en/site/index.html).


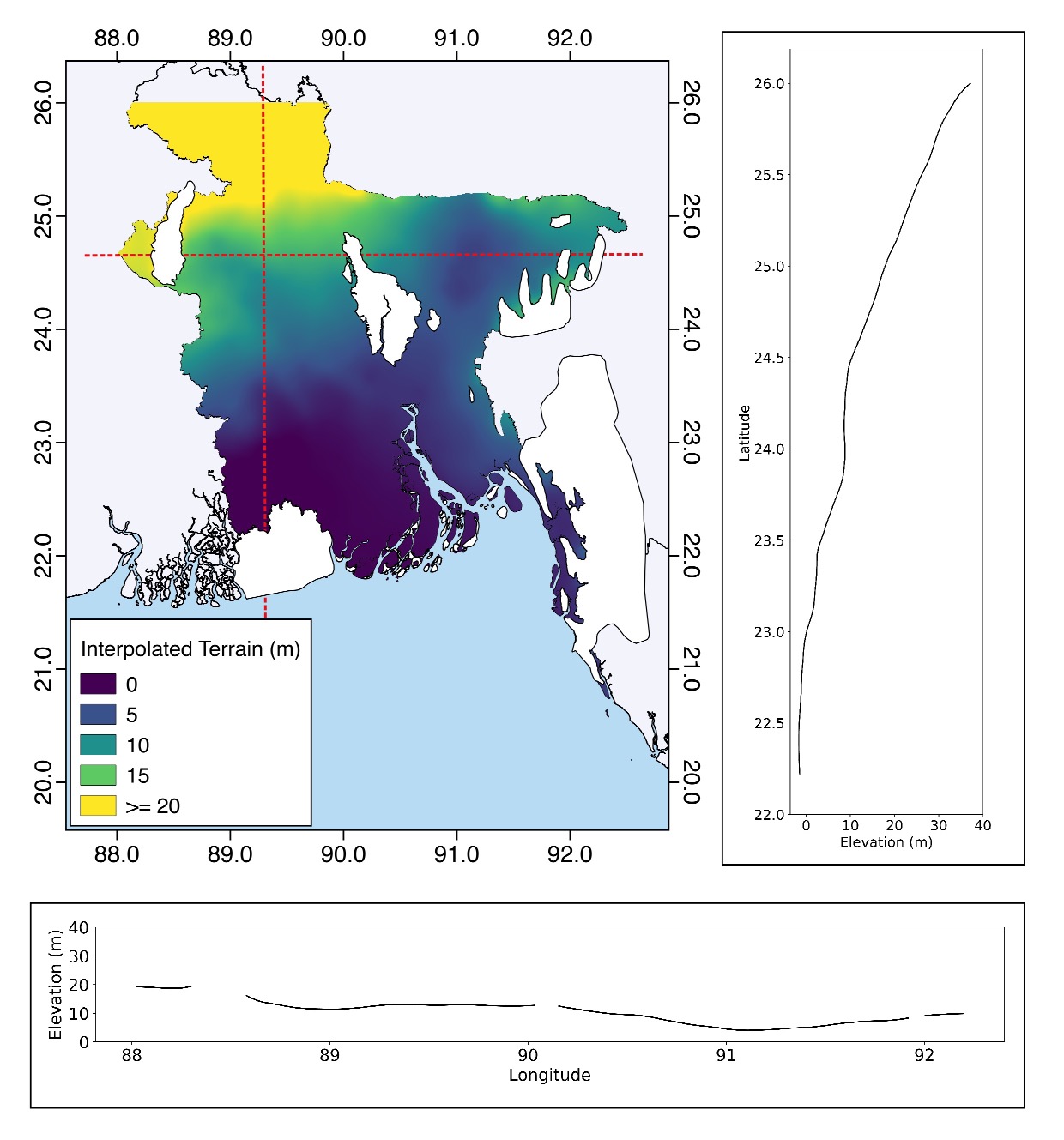


Figure S2. Interpolated Digital Terrain Model (DTM) created from the TanDEM-X DEM using ground control points. Red dashed lines are elevation profiles. Areas of elevated terrain and continuous forest are masked out. Map made with QGIS 2.8 (https://www.qgis.org/en/site/index.html).

Table S1. Confusion Matrix for the Bangladesh tree map for the classes of “tree” and “non-tree”

|  | Non-Tree | Tree | N | user accuracy (%) |
| --- | --- | --- | --- | --- |
| Non-Tree | 3724 | 276 | 4000 | 93.1 |
| Tree | 406 | 3594 | 4000 | 89.9 |
| N | 4130 | 3870 | 8000 |  |
| producer accuracy (%) | 90.2 | 92.9 |  | 91.5 |


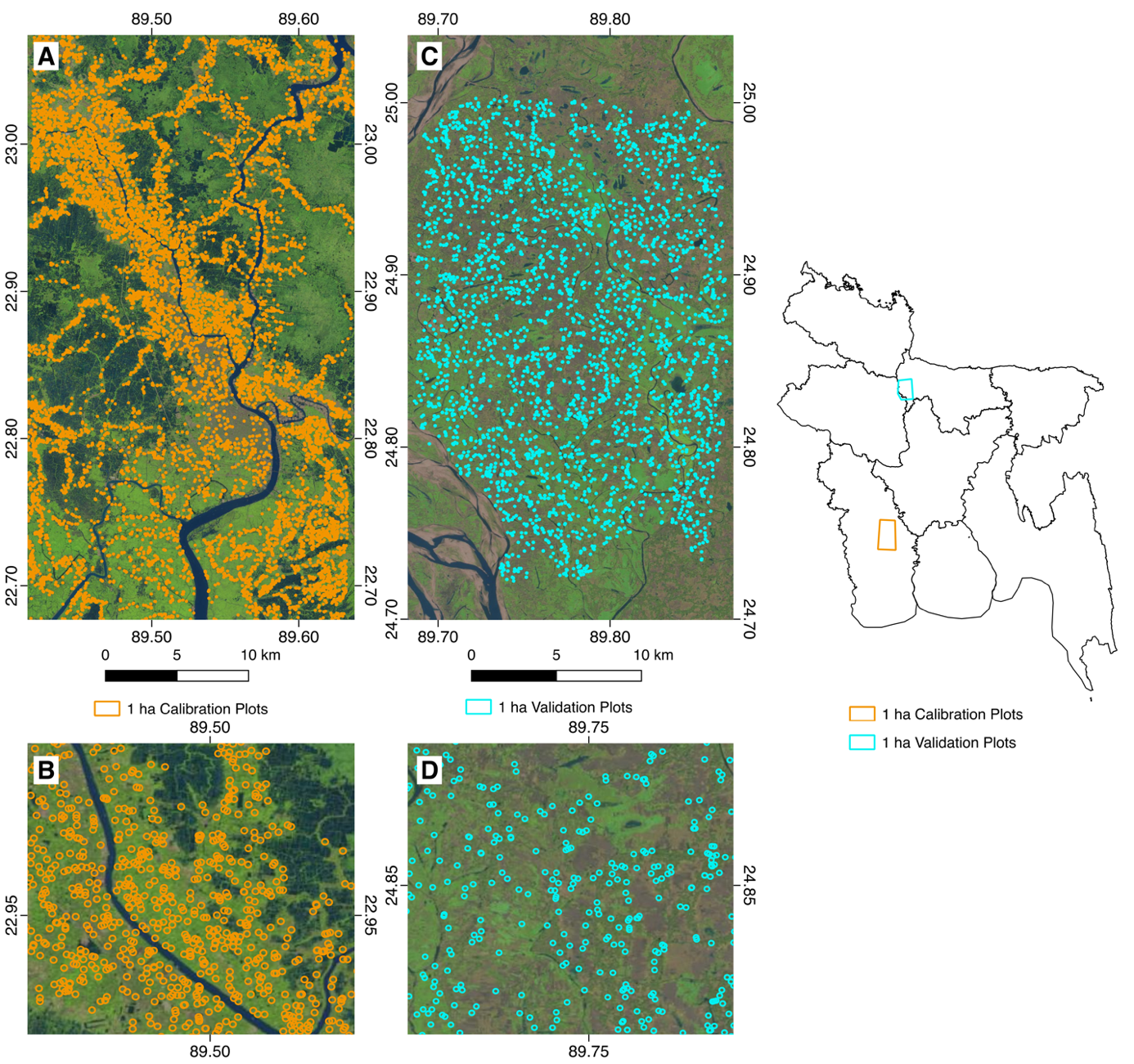


Figure S3. Location and distribution of calibration (A/B) and validation (C/D) points for modelling tree height. Maps made with QGIS 2.8 (<https://www.qgis.org/en/site/index.html>).


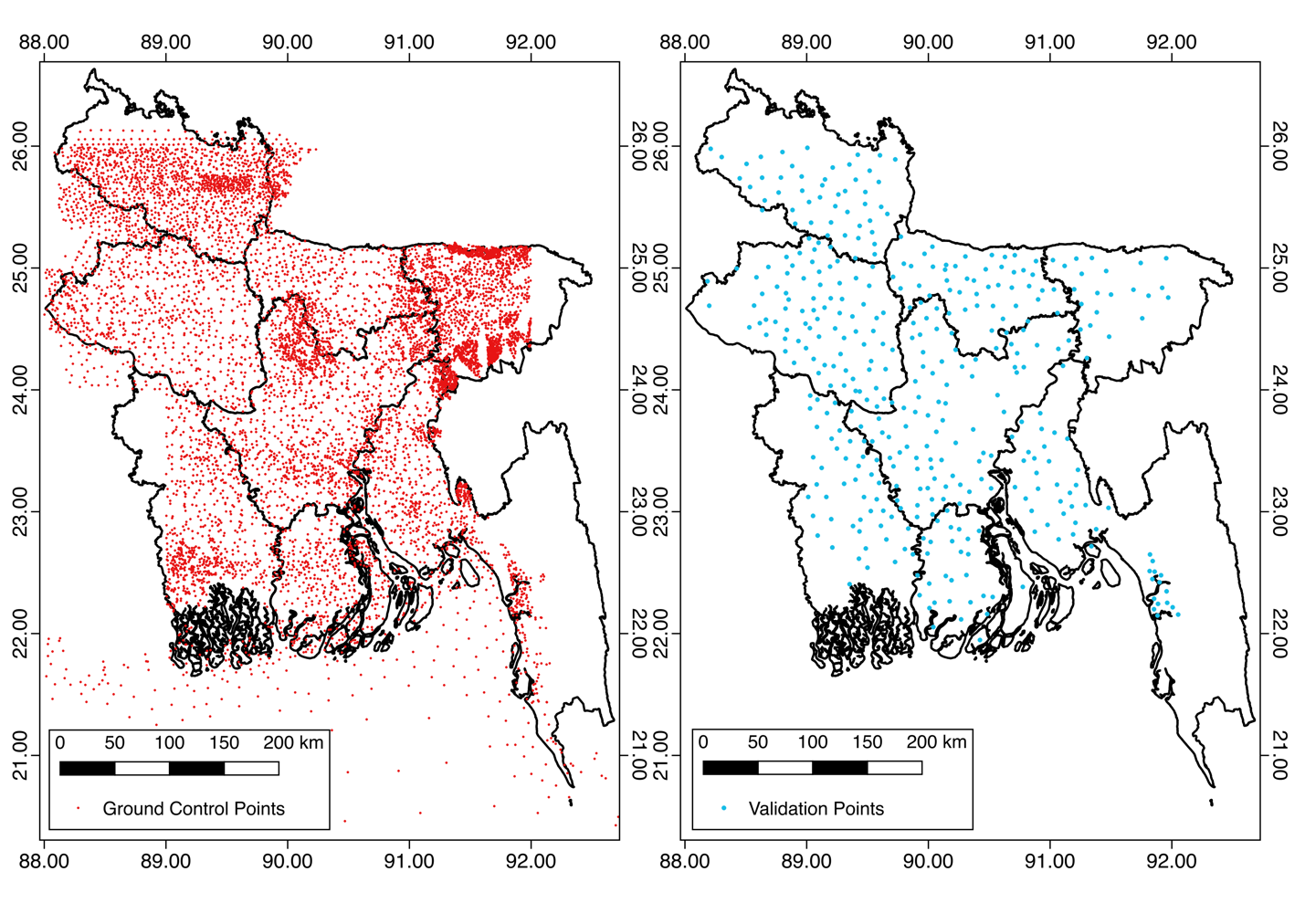


Figure S4. Distribution of Ground Control Points (GCPs) for generating the bare earth DSM (left) and independent validation points used to determine agreement with DTM (right). GCPs were collected only where the ground surface could be determined. No GCPs could be collected in some locations due to canopy cover but were masked out during the tree height analysis. Maps made with QGIS 2.8 (https://www.qgis.org/en/site/index.html).


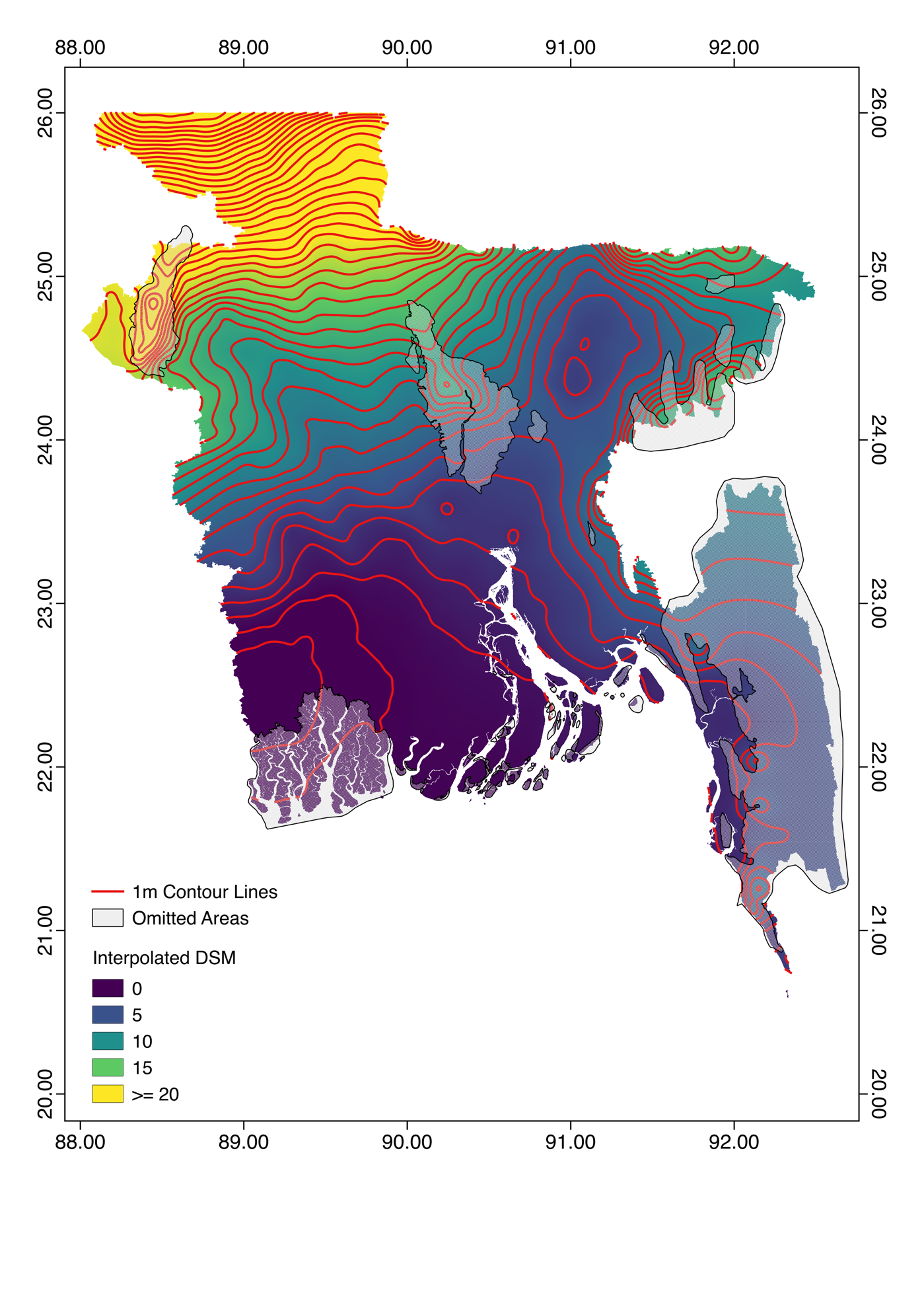


Figure S5. Contour lines for bare earth DEM at an interval of 1 m. Areas of continuous forest and elevated terrain (Omitted Areas) were interpolated but masked out of subsequent analysis as the accuracy of the surface could not be determined at these locations.
